# Evaluation of blood, buccal swabs, and hair follicles for DNA profiling technique using STR markers

**DOI:** 10.3325/cmj.2015.56.239

**Published:** 2015-06

**Authors:** Garima Chaudhary, T D Dogra, Anupuma Raina

**Affiliations:** 1 Department of Forensic Medicine, All India Institute of Medical Sciences, New Delhi, India; 2 SGT Group of Institutions, Budhera, Gurgaon, Haryana, India

## Abstract

**Aim:**

To study the short tandem repeat (STR) pattern of DNA from the blood, buccal swabs, and hair follicles of the recipients of allogenic hematopoietic stem cell transplantation to examine whether these tissues contain donor derived cells.

**Methods:**

The study enrolled 25 patients who sustained engraftment. Peripheral blood, buccal swabs, and hair follicles were collected on days 21-30, 90, and 180 after transplantation and the chimeric status of the recipients was evaluated.

**Results:**

Donor derived cells existed in the blood and buccal swabs, but not in hair follicles, which can be used to obtain the pre-transplant sample of the recipient after transplant.

**Conclusion:**

Peripheral blood and buccal swab do not serve as a reliable source of recipient’s origin for DNA analysis of individuals who underwent allogenic hematopoietic stem cell transplantation at least within 6 months after transplant.

Chimerism is an exceptional immunogenetic condition, characterized by the presence of cell populations originating from two different individuals. These cells could be derived from a fetal maternal transfusion, blood transfusion, or after allo- hematopoietic stem cell transplantation (HSCT) or mobilized peripheral blood stem cell transplantation (PBSCT) (1). BMT/PBSCT has been widely accepted as a convincing life-saving treatment modality for various malignant and non-malignant hematologic diseases. Chimerism analysis is an important tool for the pre-transplant surveillance of engraftment and offers the possibility to realize impending graft acceptance or rejection (2). Besides clinical cases, it is also important in forensic analysis.

Polymorphic STR analysis is presently the most common analytical method employed for forensic identity testing (3), since it is more sensitive than analysis with any other polymorphic markers and allows unambiguous assignment of alleles. Blood, buccal swabs, and hair follicles are among the most important and common biological samples used for DNA profiling as they carry high evidential value (4). However, these samples may lead to erroneous interpretations of results when the person being analyzed is a genetic chimera, since this condition has the potential to change the genetic makeup of the individuals.

Many studies showed that blood was not a suitable substrate for personal identification due to the presence of donor cells in the recipient blood cells (5,6). Along with blood, buccal swabs and saliva are also reported to show conversion to donor type (7-12). Other commonly encountered biological sources for forensic DNA profiling, especially in rape cases, are fingernails and hair follicles. However, there were studies showing donor chimerism in these sources as well (13-16).

Various studies have been conducted using different markers to evaluate the percentage of donor derived cells in different biological samples of recipients but there is no clear indication about the informativity of STR markers used for forensic purpose in chimerism analysis. Thus, the aim of this study was to evaluate the chimeric level in recipient blood, buccal swabs, and hair follicles at different time intervals after allo-BMT or PBSCT with the markers that are being extensively used in forensic DNA analysis.

## Materials and methods

Twenty five patients who had received allo-HSCT or PBSCT for various hematological disorders were recruited in 2010 from the department of Haematology, All India Institute of Medical Sciences (Table 1). The present study was reviewed and approved by the ethics committee of this institute (IESC/T-302/2010 of Nov 2, 2010). Written consent was obtained from every participant prior to the sampling.

**Table 1:** Quantitative summary of patients’ clinical data

Category	n	%
**Number of recipients**		
Total	25	100.00
Male	19	76.00
Female	6	24.00
**Number of samples**		
Buccal swab	75 samples	
Blood	75 samples	
Hair root	75 samples	
**Recipient /donor relationship**		
Related	25	100.00
Not related	0	0.00
**Sex match with donor**	13	52.00
**Sex mismatch with donor**	12	48.00
**Disease**		
Severe aplastic anemia	13	52.00
Thalassemia	6	24.00
Myelogenous leukemia (acute myeloid leukemia/chronic myeloidleukemia	3	12.00
Acute mixed phenotypic leukemia	2	8.00
Red blood cell aplasia	1	4.00
**Origin of stem cells**		
Peripheral blood	18	72.00
Bone marrow	7	28.00

Donor and pre-transplant recipient’s blood samples were collected for the reference material. Pre-transplant buccal swabs and hair follicles were collected to rule out the possibility of natural chimerism. All the peripheral blood samples from donors and recipients were collected in 5-mL EDTA coated tubes. Buccal swabs from recipients were collected from the both sides of the oral cavity on cotton sticks and kept frozen until the DNA extraction. Before taking buccal swab samples, patients were counseled to refrain from food and drink and maintain high level of oral hygiene, avoiding any possible contamination at least for one hour prior to the sampling. 5-8 hair strands along with root were plucked from the scalp. Presence of hair bulb was visually confirmed. Long hairs were cut to around 2 cm including the root, and distal parts were discarded.

Peripheral blood, buccal swabs, and hair follicles from the recipients were collected on days 21-30, 90, and 180 after transplantation. Genomic DNA from all the samples was isolated using organic extraction method (17). Quality and quantity of extracted genomic DNA was evaluated by agarose gel electrophoresis and UV spectrophotomety (Nanodrop, Thermospectronic, Rochester, NY, USA). Amplification of extracted DNA was carried out using AmpFlSTR identifiler kit (Applied Biosystems, Foster City, CA, USA), which co-amplifies 15 loci (D8S1179, D21S11, D7S820, CSFIPO, D3S1358, THO1, D135317, D16S539, D2S1338, D19S433, vWA, TPOX, D18S51, D5S818, FGA) and the sex determining marker, amelogenin. The amplified DNA was analyzed by ABI 3100 genetic analyzer and genotype software version 3.2 (Applied Biosystems). Three controls (Inbuilt house kit and negative one) were used to maintain the sensitivity of the analysis. Donor chimerism was calculated according to peak area of the donor and the recipient allele (18). The chimerism percentage was calculated for every possible combination: type I – by considering only alleles not shared between recipient and donor; type II – by considering at least one shared and one unshared allele between recipient and donor; type III – by considering alleles common between recipient and donor. Type III was considered to be non- informative and was excluded from chimerism percentage calculation. Allele bands shorter than 4bp, corresponding to the main allele, were considered as stutter bands and were excluded from chimerism calculations. Samples with more than 90% chimerism were classified as complete chimerism (100%Ch) and samples with less than 10% chimerism were classified as no chimerism (0%Ch).

### Statistical analysis

Data are presented as mean and standard deviation. Wilcoxon signed-rank test was used to compare the chimerism levels of different biological specimens at different time intervals. Paired *t* test was used to compare the groups of three different biological samples, ie, blood, buccal swab, hair follicle, collected at three different time intervals (21-30 days, 90 days, and 180 days post transplant). *P* < 0.05 was considered as the significance level. AdaMSoft software was used for statistical analysis.

## Results

**Figure 1:**
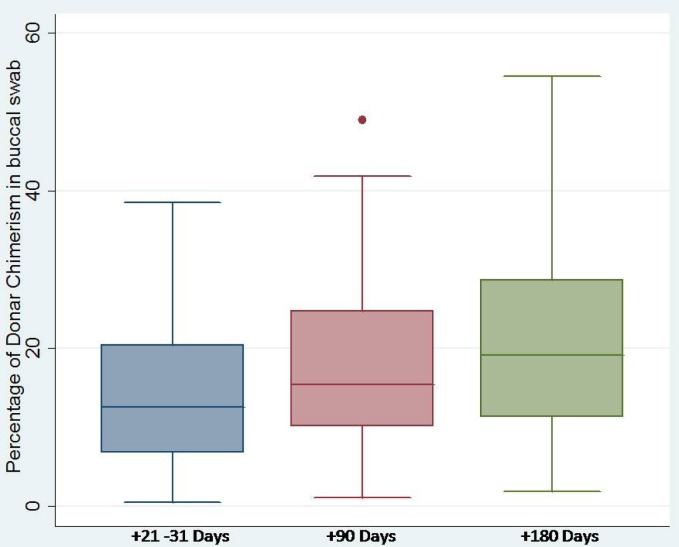
Percentage of donor chimerism in buccal swabs at different post transplant intervals in all the patients (n=25), *P*=0.001.

**Figure 2:**
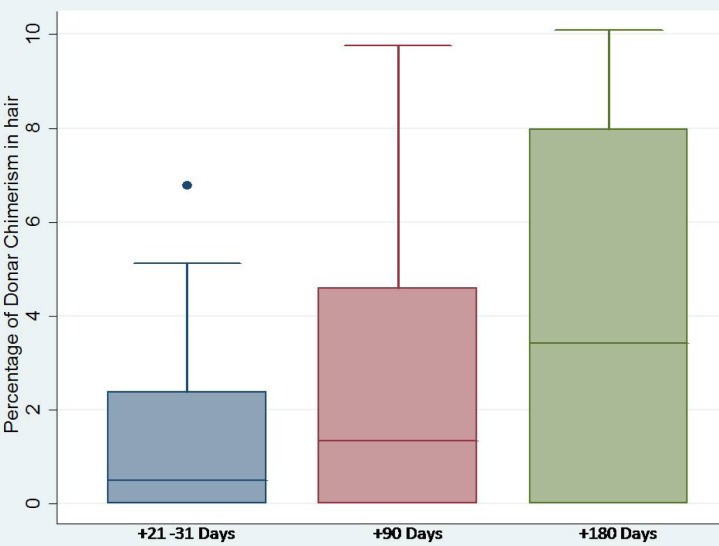
Percentage of donor chimerism in hair follicle samples at different post transplant intervals in all the patients (n=25), *P*=0.001.

19 blood samples showed complete donor chimerism (100%), 3 patients (unique patient number [UPN] 10, 16, 19) showed chimerism within the range of 92.4%-100%, and other 3 patients (UPN 13, 14, 24) showed mixed chimerism within the range of 46.9%-97% at various time points (Table 2). Buccal swabs showed donor chimerism in all the samples at all time points. 8 showed donor chimerism below 10% (UPN 9,12,14,15,17,18, 21, and 24). The majority of hair follicle samples showed donor chimerism (18 of 25), but in none chimerism was above 10%. Seven patients (UPN-5, 9, 14, 15, 18, 21, 24) showed no chimerism and remained completely of recipient type. 3 patients (UPN 1, 6, 12) showed donor chimerism below 1% and 15 patients showed donor chimerism below 10% at all the time intervals (Figure 1,2; Table 3). There was a significant change in the percentage of donor chimerism among the groups of the biological samples (peripheral blood, buccal swab, hair follicle) in recipient samples at every time point analyzed post transplant (*P* = 0.0001). With time, gradual increase in the percentage of donor chimerism was observed. No correlation of chimerism level with patients’ age and no correlation between different groups of biological samples and donor chimerism and different time intervals post transplant (*P* > 0.05) (Figure 1 and 2) was observed.

**Table 2:** Characteristics and percentage chimerism of different biological samples at different time intervals

Unique patient number	Sex/age at HSCT	Donor sex	Diagnosis	Type of transplantation	+21 to +31 days			+3 months			+6 months		
					blood percent donor type	buccal swab percent donor type	hair percent donor type	blood percent donor type	buccal swab percent donor type	hair percent donor type	blood percent donor type	buccal swab percent donor type	hair percent donor type
1	M/11	M	Thalassemia	BMT	99.75	16.72	0	98.32	24.00	0	90.58	28.72	1.08
2	M/3	F	CDA with thalassemia	BMT	100	23.00	1.34	100	25.98	2.16	100	27.00	4.45
3	M/39	F	AML	PBSCT	100	12.3	0.56	100	15.4	1.21	100	18.3	2.00
4	M/18	F	Severe aplastic anemia	PBSCT	100	12.60	1.98	100	14.33	2.32	100	24.97	3.67
5	M/35	F	Severe aplastic anemia	PBSCT	100	9.2	0	100	10.1	0	100	11.3	0
6	M/11	M	Aplastic anemia	PBSCT	100	10.5	0	100	11.6	0.67	100	12.3	0.98
7	F/45	F	Severe aplastic anemia	PBSCT	100	15.9	2.38	100	16.4	2.62	100	19.2	3.54
8	M/8	F	Thalassemia	BMT	100	20.5	4.38	100	25.1	5.12	100	29.7	8.00
9	M/31	M	Refractive pure RBC aplasia	PBSCT	100	1.23	0	100	2.45	0	100	2.98	0
10	F/21	F	Severe aplastic anemia	PBSCT	92.4	20.09	0.49	96.41	24.7	1.33	97.5	32.00	9.50
11	M/35	**F**	Severe aplastic anemia	PBSCT	100	14.2	4.25	100	17.4	5.87	100	25.7	8.00
12	M/41	M	Mixed phenotypic acute leukemia	PBSCT	100	2.56	0	100	3.59	0	100	5.36	0.67
13	M/8	F	Very severe aplastic anemia	BMT	87	35.6	4.6	91	41.8	8.65	97	53.6	9,12
14	F/3	F	Thalassemia	BMT	54.6	1..5	0	55.1	2.86	0	57	4.00	0
15	M/21	F	Very severe aplastic anemia	PBSCT	100	6.73	0	100	10.6	0	100	12.3	0
16	M/15	M	Severe aplastic anemia	PBSCT	93	38.5	6.78	95	48.9	9.76	99	54.5	10.09
17	M/22	M	Very severe aplastic anemia	PBSCT	100	5.6	0	100	9.4	1.23	100	12.6	3.41
18	M/32	M	CML	PBSCT	100	7.54	0	100	10.7	0	100	11.2	0
19	M/28	F	Severe aplastic anemia	PBSCT	97	15.6	2.31	99	17.5	4.59	100	24.8	6.23
20	F/42	F	Acute myeloid leukemia	PBSCT	100	22.1	5.12	100	24.00	6.00	100	25.1	9.49
21	M/42	M	Aplastic anemia	PBSCT	100	2.56	0	100	5.34	0	100	7.8	0
22	M/8	F	Thalassemia major	BMT	100	10.4	0.23	100	12.6	1.98	100	15.5	2.00
23	F/39	M	CML	PBSCT	100	28.9	3.24	100	36.7	5.23	100	45.8	7.98
24	F/12	F	Thalassemia major	BMT	55.00	0.5	0	51.2	1.11	0	46.9	1.9	0
25	M/17	F	Severe aplastic anemia	PBSCT	100	21.6	0.76	100	26.8	2.89	100	30.4	5.71
		**Mean** **(range)**			**95.15** **(54.6-100)**	**14.76** **(0.5-38.5)**	**1.53** **(0-6.78)**	**95.44** **(51.2-100)**	**17.57** **(1.11-48.9)**	**2.46** **(0-8.65)**	**95.51** **(46.9-100)**	**21.48** **(1.9-54.5)**	**3.61** **(0-10.09)**

*BMT – bone marrow transplantation; PBSCT – peripheral blood stem cell transplantation; CDA – congenital dyserythropoietic anemia ; AML – acute myeloid leukemia; RBC – red blood cells.

**Table 3:** Mean, standard deviation, and p50 value of peripheral blood, buccal swabs, and hair follicles at different time intervals post transplant for all 25 patients

Sample	Mean	Standard deviation	p50
**Blood (+21-31 d)**	95.15	12.554	100 (54.6-100)
**Blood (+3 mo)**	95.44	12.910	100 (51.2-100)
**Blood (+6 mo)**	95.51	13.341	100 (46.9-100)
**Buccal swab (+21-31 d)**	14.23	10.340	12.6 (0.5-38.5)
**Buccal swab (+3 mo)**	17.57	12.310	15.4 (1.11-48.9)
**Buccal swab (+6 mo)**	21.48	14.569	19.2 (1.9-54.5)
**Hair follicle (+21-31 d)**	1.53	2.040	0.49 (0-6.78)
**Hair follicle (+3 mo)**	2.46	2.869	1.33 (0-9.76)
**Hair follicle (+6 mo)**	3.83	3.698	3.41 (0-10.09)

## Discussion

A few cases of chimerism have been reported in forensic context but this is still an under-reported genetic peculiarity (19). During the medico legal investigations this information is often not available. Our results suggest that all patients who had a history of allo-BMT/PBSCT showed complete donor profile in blood, with few exceptions and mixed chimerism in buccal swabs and hair follicles.

For all the hair samples, percentage of donor chimerism was between 0%-10% hence this could be classified as “no chimerism.” The small percentage of donor DNA observed in hair follicle samples could have been the result of contamination with leukocytes because blood in traces could have come out along with hair follicle. Although proper care was taken to remove the possible contamination from the plucked hair follicle, the possibility of contamination cannot be ruled out completely. Also, the technique we used is very sensitive to such contamination; the sensitivity of Identifiler® kit is given in terms that full profile can be obtained from 2 ng down to 0.25 ng of human genomic DNA (Applied Biosystem, User manual).

The genotype of buccal swabs in all the patients showed donor chimerism. There are few possible explanations for the presence of high percentage of donor DNA in buccal swabs. During the collection of buccal swabs epithelial cells can be contaminated with saliva. Since granulocytes and lymphocytes are trafficking through the normal human mucosa and can be found in saliva, after successful transplantation these granulocytes and lymphocytes in recipient are derived from the donor. There is highly divergent information on the number of leukocytes in the normal saliva, ranging from 2-136 000 cells/mL^2^ to 1.1 × 10^6^ cells/mL^2^ in a patient with inflammation of the oral cavity (20). In order to remove as many non epithelial cells as possible from the buccal swab before collection, patients were asked to thoroughly rinse their mouth with water but still there was a possibility of mixing of leukocytes with epithelial cells. Similarly, it was found that mouthwash sample of patients who underwent bone marrow transplant contained high amount of donor DNA and was sometimes almost completely derived from the donor (7). Further, when epithelial cells were separated out from the mouthwash sample and analyzed for donor chimerism percentage, it was found that epithelial cells served as a good source to obtain recipient pre-transplant DNA profile (8). This study observed that 8 out of 25 patients showed lower percentage of donor chimerism (below 10%), while UPN 16 showed high donor chimerism (54.5%). It is still not clear which factor defines the variable number of donor cells present in a buccal swab.

Jacewicz et al (15) found donor derived male DNA in the range of 0.96-19.16 ng/µL in epithelial cells of female recipients who received allo-HSCT. According to them, the possible reason for the presence of donor chimerism is the “stem cell plasticity phenomenon” (21), ie, the ability of adult stem cells to cross lineage barrier and differentiate into cells outside their own tissue. Similarly, Berger et al (12) found donor DNA in every sample of buccal swab of adult recipients of allo-HCT and concluded that the presence of donor DNA in recipient buccal swab of such patients is a rule rather than the exception. They could not explain the biological cause of donor DNA presence in buccal epithelia of the recipient but believed it unlikely that it was caused by the migration of leukocytes in to the buccal epithelia as the collected buccal swab exclusively consisted of epithelial cells with no contamination of saliva.

Various studies showed the presence of donor cells in different tissues of recipient’s body. Imanishi et al (13) demonstrated the existence of donor cells in fingernails of recipients of allo-HCT transplant patients. Due to the myeloblative conditioning regimen of transplanted patients, their epithelial stem cells got damaged to a significant extent, which led to the transient growth retardation. This contributes to de-differentiation of donor hematopoietic stem cells into non-hematopoietic tissue of recipient.

After engraftment, production of donor cells from bone marrow in allo-transplanted recipients is an ongoing process. These donor cells undergo their programmed death, apoptosis, and release donor DNA packaged into apoptotic bodies (22-25). Although foreign DNA is normally cleared up (26), the excessive amount of released donor-derived genetic material is horizontally transferred from apoptic hematopoietic cells to the cytoplasm and nucleus of epithelial cell lines through phagocytosis of apoptic bodies and is integrated within the recipient’s genome, resulting in DNA chimerism (27). Furthermore, the incorporation of the foreign DNA into the host genome could result in physical rearrangement at the site of integration, including point mutations, deletions, interruptions of coding sequence, and chromosomal breakages.

After allo-HSCT, epithelial tissues also become injured through preparative regimen and are then potentially attacked by allo-reactive T cells. The net effect of this allo-antigenic reaction is tissue stress and apoptosis, which is known as graft vs host disease. Chronic stress due to interaction of donor derived lymphocytes with host epithelium in the biological chimera may cause genomic alteration (28). Hence, the development of epithelial cells with donor derived genotype and the accumulation of genomic alterations in the epithelial tissue are the recognized phenomena occurring in the recipient after allo-HSCT, explaining the presence of donor derived DNA in recipient epithelial cells.

We observed a difference in donor chimerism among the individuals in all the three samples (blood, buccal swabs, and hair follicles) and interestingly every individual showed difference in chimerism level at different post transplant intervals. The effect of post transplant intervals was also analyzed on the chimeric levels in every patient. With the increase in post transplant intervals, chimeric level increased in every patient but there was no significant co-relation between age and sex with chimerism percentage. The limitation of this study is that we performed follow up of patients up to 180 days post transplant due to the time constraints.

In summary, blood and buccal swabs are not useful to get the patient’s pretransplant or true genotype profile as these samples are not devoid of donor derived cells. For hair samples, no donor chimerism was observed so they can be a reliable biological source for personal identification using DNA profiling technique. Also, great care must be taken to avoid possible contamination while collecting the samples. The findings of this study are useful as supportive data for forensic DNA profiling.
