# Evaluation of Blood, Buccal Swabs, And Hair Follicles for DNA Profiling Technique Using STR Markers

**DOI:** 10.3325/cmj.2015.56.511

**Published:** 2015-10

The authors of the article Evaluation of Blood, Buccal Swabs, And Hair Follicles for DNA Profiling Technique Using STR Markers (Croat Med J. 2015;56(3):239-45) have informed the Journal that two authors, Uma Kanga and Tulika Seth, were inadvertently omitted from the published version of their manuscript due to a misunderstanding regarding the authorship criteria. The initial authorship list read as follows:

Chaudhary G, Dogra TD, Raina A. Evaluation of blood, buccal swabs, and hair follicles for DNA profiling technique using STR markers. Croat Med J. 2015 Jun;56(3):239-45.

The byline has been corrected in the PDF and HTML versions of the Article and is as follows:

Chaudhary G, Seth T, Kanga U, Dogra TD, Raina A. Evaluation of blood, buccal swabs, and hair follicles for DNA profiling technique using STR markers. Croat Med J. 2015 Jun;56(3):239-45.

The authors declared no conflicts of interest.

